# DHODH Drives Sunitinib Resistance Via a Non‐Enzymatic Mechanism by Inhibiting TRIM28 Ubiquitination and Consequent VEGFA Activation in RCC

**DOI:** 10.1002/advs.76266

**Published:** 2026-06-23

**Authors:** Shijie Qian, Shihang Pu, Zhipeng Xu, Yiheng Ding, Ling Jin, Baochao Li, Qi Li, Xiaohai Xi, Zengjun Wang, Jiajun Xing, Aiming Xu

**Affiliations:** ^1^ Department of Urology First Affiliated Hospital of Nanjing Medical University Nanjing China

**Keywords:** DHODH, renal cell carcinoma, sunitinib resistance, ubiquitination

## Abstract

Sunitinib, a tyrosine kinase inhibitor, exerts its crucial therapeutic effect in renal cell carcinoma (RCC) primarily by inhibiting tumor angiogenesis and cellular proliferation. However, despite its initial efficacy, therapeutic resistance invariably develops in many RCC patients, underscoring the urgent need for novel strategies to overcome this limitation. We constructed patient‐derived xenograft (PDX) models using surgically resected RCC tissues and subsequently established stable sunitinib‐resistant and sunitinib‐sensitive PDX models. Proteomic profiling was performed to identify key regulators involved in sunitinib resistance. Mechanistic studies were conducted to investigate the molecular interactions among DHODH, TRIM28, and VEGFA. The therapeutic potential of lisaftoclax was evaluated in combination with sunitinib. Proteomic profiling identified DHODH as a key regulator in the development of sunitinib resistance in RCC. Mechanistically, DHODH competes with the E3 ligase TRIM37 for binding to TRIM28, thereby stabilizing TRIM28 by inhibiting its ubiquitination. TRIM28 subsequently activates VEGFA transcription, which promotes sunitinib resistance in RCC. Lisaftoclax, a small‐molecule inhibitor that disrupts the DHODH‐TRIM28 interaction, potentiates sunitinib efficacy and exerts a synergistic therapeutic effect. Collectively, our findings identify DHODH as a critical therapeutic target for overcoming sunitinib resistance in RCC and provide a novel strategy for the treatment of RCC.

## Graphical abstract

This non‑enzymatic function of DHODH drives sunitinib resistance by competing with TRIM37 to block TRIM28 ubiquitination, thereby stabilizing TRIM28 and activating VEGFA transcription. Disrupting the DHODH–TRIM28 interaction with lisaftoclax restores drug sensitivity.

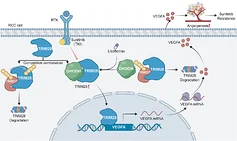

**Abbreviations**
ATCC: american type culture collection
CCK‐8: cell counting kit‐8
RCC: renal cell carcinoma
CHX: cycloheximide
Co‐IP: co‐immunoprecipitation
CQ: chloroquine
CUT & RUN: cleavage under targets and release using nuclease
DEGs: differentially expressed genes
DHODH: dihydroorotate dehydrogenase
dTMP: deoxythymidine monophosphate
dUMP: deoxyuridine monophosphate
DMEM: Dulbecco's modified eagle medium
DMSO: dimethyl sulfoxide
DSBs: DNA double‐strand breaks
ELISA: enzyme‐linked immunosorbent assay
FBS: fetal bovine serum
Flt3: fms‐related receptor tyrosine kinase 3
FMN: flavin mononucleotide
GO: gene ontology
GPX4: glutathione peroxidase 4
HIFs: hypoxia‐inducible factors
HP1: heterochromatin protein 1
HREs: hypoxia‐response elements
HUVECs: human umbilical vein endothelial cells
IC50: half‐maximal inhibitory concentration
IHC: immunohistochemistry
IP‐MS: immunoprecipitation‐based mass spectrometry
KAP1: KRAB‐associated protein 1
KD: knockdown
KEGG: kyoto Encyclopedia of Genes and Genomes
NF‐κB: nuclear factor kappa B
NSG: NOD‐scid‐IL2rg^−^/^−^ mice
O‐GlcNAcylation: O‐linked β‐N‐acetylglucosaminylation
OGT: O‐GlcNAc transferase
ORF: open reading frame
PBS: phosphate‐buffered saline
PDGF: platelet‐derived growth factor
PDO: patient‐derived organoid
PDX: patient‐derived xenograft
qRT‐PCR: quantitative reverse transcription PCR
RCC: renal cell carcinoma
RNA‐seq: RNA sequencing
ROS: reactive oxygen species
SUMO2: small ubiquitin‐like modifier 2
TF: transcription factor
TMAs: tissue microarrays
TRAF6: TNF receptor associated factor 6
TRIM28: tripartite motif containing 28
TRIM37: tripartite motif containing 37
VEGF: vascular endothelial growth factor
VHL: von Hippel‐Lindau tumor suppressor

## 1 Introduction

Renal cell carcinoma (RCC) is a prevalent malignancy, accounting for an estimated lifetime risk of 2.3% in men and 1.3% in women in the United States [1]. The three main histological subtypes of RCC are clear cell (75%–80%), papillary (10%–15%), and chromophobe (5%) [2]. Clear cell RCC (ccRCC) is strongly associated with loss of the von Hippel‐Lindau (VHL) tumor suppressor gene, occurring in up to 90% of cases [3]. VHL inactivation typically results from mutation and/or deletion on chromosome 3p and disrupts the degradation of hypoxia‐inducible factors (HIFs), leading to constitutive activation of hypoxia‐responsive pathways, enhanced angiogenesis, and highly vascular tumors prone to hemorrhage [4, 5]. Clear cell RCC also harbors recurrent mutations in several other genes, notably PBRM1 (29–41% of tumor samples), SETD2 (8–12%), BAP1 (6–10%), KDM5C (4–7%), and MTOR (5–6%) [6, 7].

For patients with metastatic, non‐operable RCC, sunitinib serves as a first‐line therapeutic agent and functions as a multi‐targeted tyrosine kinase inhibitor [8]. Its mechanism of action involves the inhibition of kinase activity in vascular endothelial growth factor receptors (VEGFR‐1 and VEGFR‐2), platelet‐derived growth factor receptors (PDGFRα and PDGFRβ), as well as c‐Kit, rearranged during transfection (RET), and fms‐related receptor tyrosine kinase 3 (Flt3) [9]. However, a substantial proportion of RCC patients develop resistance to sunitinib after a median of 6 to 15 months of treatment, leading to disease progression [10]. Multifactorial mechanisms have been identified, including upregulation of pro‐angiogenic pathways, alterations in the tumor microenvironment, induction of epithelial‐mesenchymal transition, epigenetic dysregulation, and inadequate target inhibition [11]. Therefore, elucidating the mechanisms of sunitinib resistance is crucial to enhance treatment efficacy and extend median survival in patients with advanced renal cell carcinoma.

Dihydroorotate dehydrogenase (DHODH), a flavin‐dependent, iron‐containing enzyme of the mitochondrial inner membrane, is essential for de novo pyrimidine synthesis [12]. In the de novo pyrimidine synthesis pathway, DHODH executes the fourth step via a redox reaction in mitochondria that converts dihydroorotate to orotate and reduces ubiquinone (CoQ) to ubiquinol (CoQH2), the latter serving as a direct substrate for respiratory complex III [13, 14]. Due to their heightened reliance on de novo pyrimidine synthesis, rapidly proliferating tumor cells are particularly vulnerable to DHODH inhibition, thus making it an effective anti‐proliferative strategy [15, 16]. Beyond its canonical role in pyrimidine synthesis, DHODH modulates diverse biological processes, including O‐GlcNAcylation, cellular senescence, and mRNA translation (which is indirectly affected via pyrimidine depletion) [17, 18, 19].

In patient‐derived xenograft (PDX) models, we observed that DHODH expression is significantly upregulated in sunitinib‐resistant tumors, implicating its potential role in therapeutic resistance—an area previously unexplored. Here we report a novel, non‐canonical mechanism wherein DHODH competitively occupies the binding site between TRIM37 and TRIM28 via direct interaction, thereby suppressing TRIM28 ubiquitination and degradation. This stabilization leads to enhanced VEGFA transcriptional activity, which mediates sunitinib resistance. Furthermore, we designed a targeted inhibitor against this interaction site and demonstrated, both in vitro and in vivo, that this inhibitor substantially reverses DHODH‐driven sunitinib resistance, offering a potential therapeutic strategy for patients with advanced renal cell carcinoma.

## 2 Materials and Methods

### 2.1 Human Tissue Specimens

All clinical specimens of RCC tumors and adjacent normal tissues were collected from the Department of Urology at the First Affiliated Hospital of Nanjing Medical University. The collection and analysis of clinical specimens were performed under the approval of the Ethics Committee of Nanjing Medical University, and written informed consent was obtained from all patients.

### 2.2 Immunohistochemistry (IHC) and Immunofluorescence (IF)

All tissue sections were routinely dewaxed and rehydrated. Antigen retrieval was conducted by microwave heating in citrate buffer (pH 9.0). Endogenous peroxidase activity was blocked by incubating sections in 0.3% H_2_O_2_ for 10 min. For immunohistochemistry (IHC), slides were incubated with primary antibody overnight at 4°C, followed by a species‐matched secondary antibody (1:200 dilution) at room temperature for 1 h. Signal detection was performed using horseradish peroxidase‐conjugated streptavidin with 3,3′‐diaminobenzidine tetrahydrochloride (DAB) as the chromogen. Finally, sections were counterstained with hematoxylin and mounted with a non‐aqueous medium.

For immunofluorescence (IF), following similar primary and secondary antibody incubation steps, nuclei were labeled with DAPI (1 µg/mL, 5 min). Coverslips were applied using an anti‐fade mounting medium to minimize fluorescence quenching.

### 2.3 Cell Lines and Culture Conditions

The human renal cell carcinoma cell lines 769‐P (CL‐0009), Caki‐1 (CL‐0052), 786‐O (CL‐0010), A498 (CL‐0254), ACHN (CL‐0021), and the human umbilical vein endothelial cells HUVECs (CP‐H082) were purchased from Procell Life Science & Technology (Wuhan, China). The human embryonic kidney cell line 293T (CVCL_0063) was purchased from American Type Culture Collection (ATCC, USA). 786‐O and 769‐P cells were cultured in RPMI‐1640 (Gibco) with 10% FBS (Gibco) and 1% penicillin‐streptomycin (Gibco). 293T cells were maintained in Dulbecco's Modified Eagle Medium (DMEM, Gibco) supplemented with 10% FBS (Gibco) and 1% penicillin‐streptomycin (Gibco). Caki‐1 cells were maintained in McCoy's 5A medium (Gibco) supplemented with 10% FBS (Gibco) and 1% penicillin‐streptomycin (Gibco). A498 and ACHN cells were maintained in MEM (Gibco) supplemented with 10% FBS (Gibco) and 1% penicillin‐streptomycin (Gibco). HUVEC cells were maintained in F12‐K (Gibco) supplemented with 10% FBS (Gibco) and 1% penicillin‐streptomycin (Gibco). Cells were incubated at 37°C in a humidified atmosphere with 5% CO_2_.

### 2.4 Generation of Sunitinib‐Resistant RCC Cell Lines and PDX Models

Cells (769‐P and Caki‐1) were intermittently exposed to increasing concentrations of sunitinib, starting at 4 µm. The drug concentration was gradually escalated to 20 µm after tolerance was confirmed. The establishment of sunitinib‐resistant cell lines (769‐P‐R and Caki‐1‐R) was verified by CCK‐8 assays under continuous sunitinib pressure.

PDX models were established using tumor tissues obtained from treatment‐naive RCC patients. Briefly, fresh tumor specimens were washed in cold PBS supplemented with 10% fetal bovine serum (FBS) and penicillin/streptomycin, then cut into 3–5 mm^3^fragments. These fragments were subcutaneously implanted into 6‐week‐old male NOD‐scid‐IL2rg^−^/^−^(NSG) mice. To induce sunitinib resistance, PDX model mice were randomized into two cohorts: one group received sunitinib (40 mg/kg/day), and the other received normal saline. Acquired resistance was evidenced by the tumor progression after an initial response.

### 2.5 Generation of RCC Patient‐derived Organoids (PDO)

Under sterile conditions, RCC tumor tissue samples were minced into approximately 1–3 mm^3^ fragments. Digestion solution was added, and the tissue was digested at 37 °C for 30 min. The mixture was filtered through a 100 µm strainer, and the filtrate was collected into a 15 mL centrifuge tube. The tube was centrifuged at 300 g for 5 min at 4 °C, after which the supernatant was removed. Matrix gel was added to the cell pellet, mixed by pipetting, and then plated. The prepared culture plate was placed in a 37 °C incubator for 60 min to allow gel polymerization, after which 500 µL of organoid culture medium was added per well for continued culture.

### 2.6 Chemicals, Plasmids, and Lentivirus

The chemicals used in this study: Sunitinib (S7781), Chloroquine (S6999), MG132 (S2619), Cycloheximide (S7418), and Lisaftoclax (S9970) were purchased from Selleck. Lipofectamine 3000 (L3000075) was purchased from Thermofisher.

Plasmids and Lentivirus were constructed by GENECHEM Biotech and Corues Biotechnology. The sequences of all short‐hairpin RNAs (shRNAs) are listed in Table S1.

### 2.7 CCK‐8 Assay

Cells were seeded at a density of 1000 cells per well in 96‐well plates and maintained in specific experimental conditions. After 24 h of culture, 10 µL of CCK‐8 reagent (Apexbio) was added to each well. The plates were then incubated at 37 °C for 1.5 h. Cell viability was monitored by measuring absorbance at 450 nm for 5 consecutive days. For drug sensitivity assays, cells were incubated with serial dilutions of sunitinib (0–32 µm) for 48 h. Cell viability was determined by CCK‐8 assay as previously described. The IC50 value was calculated from dose‐response curves using nonlinear regression.

### 2.8 Colony Formation Assay

Cells were seeded at a density of 1000 cells per well in 6‐well plates and maintained in specific experimental conditions. The plates were incubated at 37°C in a humidified atmosphere with 5% CO_2_ for 10–14days. Colonies were fixed with 4% paraformaldehyde, followed by staining with 0.5% crystal violet (Beyotime) for 30 min. Colonies containing more than 50 cells were counted with ImageJ.

### 2.9 Tubule Formation Assay

HUVECs were co‐cultured with treated cells for 24 h. 24‐well plates were precoated with 100 µL of Matrigel (Corning, USA) per well and incubated at 37 °C for 60 min to allow polymerization. Subsequently, HUVECs were resuspended in conditioned medium at a density of 1.5×10^5^ cells/well in a final volume of 200 µL, seeded onto the polymerized Matrigel, and incubated at 37°C for 4–6 h. Tube formation was assessed by imaging randomly selected fields under an inverted microscope (Leica). The total number of Nb nodes was then quantified with ImageJ software.

### 2.10 Apoptosis Assay

Apoptosis was induced as needed, and untreated cells were used as negative controls. Cells were harvested, rinsed with PBS, and stained with Annexin V‐APC and 7‐AAD using an apoptosis detection kit (KeyGEN BioTECH, KGA1106) according to the manufacturer's protocols. Following 15–20 min of incubation in the dark, samples were subjected to flow cytometric analysis.

### 2.11 Orthotopic and Subcutaneous Xenograft Tumor Models

Orthotopic xenografts were established by injecting 5 × 10^5^ 769‐P cells under the renal capsule of nude mice. Subcutaneous xenografts were established by injecting 1 × 10^6^ 769‐P cells into the flanks of nude mice. Four weeks post‐implantation, mice were treated daily by oral gavage with either sunitinib (40 mg/kg) or DMSO, and mice were sacrificed at the end of the second month.

### 2.12 Cleavage Under Targets and Release Using Nuclease (CUT&RUN) Assay

The CUT&RUN detection was performed using the CUT&RUN Assay Kit (Vazyme) according to manufacturer instructions, and qRT‐PCR was performed to verify the TRIM28‐binding sites of VEGFA

### 2.13 Dual‐luciferase Reporter Assay

Wild‐type or mutated the TRIM28‐binding sites of VEGFA were co‐transfected into HEK‐293T cells along with either the control vector or TRIM28 overexpression plasmids. After 48 h of incubation, luciferase activities were measured using a Dual‐Luciferase Reporter Assay System (Vazyme) according to manufacturer instructions. Firefly luciferase activity was normalized to Renilla luciferase activity to account for variations in transfection efficiency. The firefly luciferase/Renilla luciferase (Fluc/Rluc) ratio was used to assess the binding affinity between TRIM28 and the corresponding VEGFA DNA fragments.

### 2.14 Quantitative Real‐Time PCR (qRT‐PCR)

Total RNA was extracted using NcmSpin RNA Quick Purification Kit (NCM Biotech, China), and complementary DNA (cDNA) was then synthesized using the All‐in‐One Ultra RT SuperMix (Vazyme, China). qRT‐PCR was performed using the One Step qRT‐PCR SYBR Green Kit (Vazyme, China) on a quantitative PCR system. All the primer sequences used in this study are provided in Table S2.

### 2.15 Western Blot Analysis

Total protein was extracted using RIPA buffer (Fude Bio, China) containing protease and phosphatase inhibitors (Fude Bio, China). The concentration of proteins was qualified by the BCA protein assay Kit (Beyotime, China). Subsequently, equal amounts of protein samples were separated by 10% SDS‐PAGE and transblotted onto nitrocellulose membranes (BioRad, Cat# 1620115). After being blocked in 5% nonfat milk for 2 h at room temperature, the members were incubated with the indicated primary antibodies overnight at 4°C. Following incubation with secondary antibody for 2 h at room temperature, protein bands were visualized using an enhanced chemiluminescence (ECL) detection kit (NCM Biotech, China) and imaged with the ECL detection system (Bio‐Rad, USA). The antibodies used in this study are listed in Table S3.

### 2.16 Statistical Analysis

Statistical analyses were performed using GraphPad Prism 9.0 (GraphPad Software, San Diego, CA, USA) or R software (version 4.2.0). The data are presented as the means±SDs. Ns, not significant; ^*^ *P* < 0.05, ^**^ *P* < 0.01, ^***^ *P* < 0.001.

## 3 Result

### 3.1 DHODH Overexpression is Associated with Sunitinib Resistance in RCC

**FIGURE 1:**
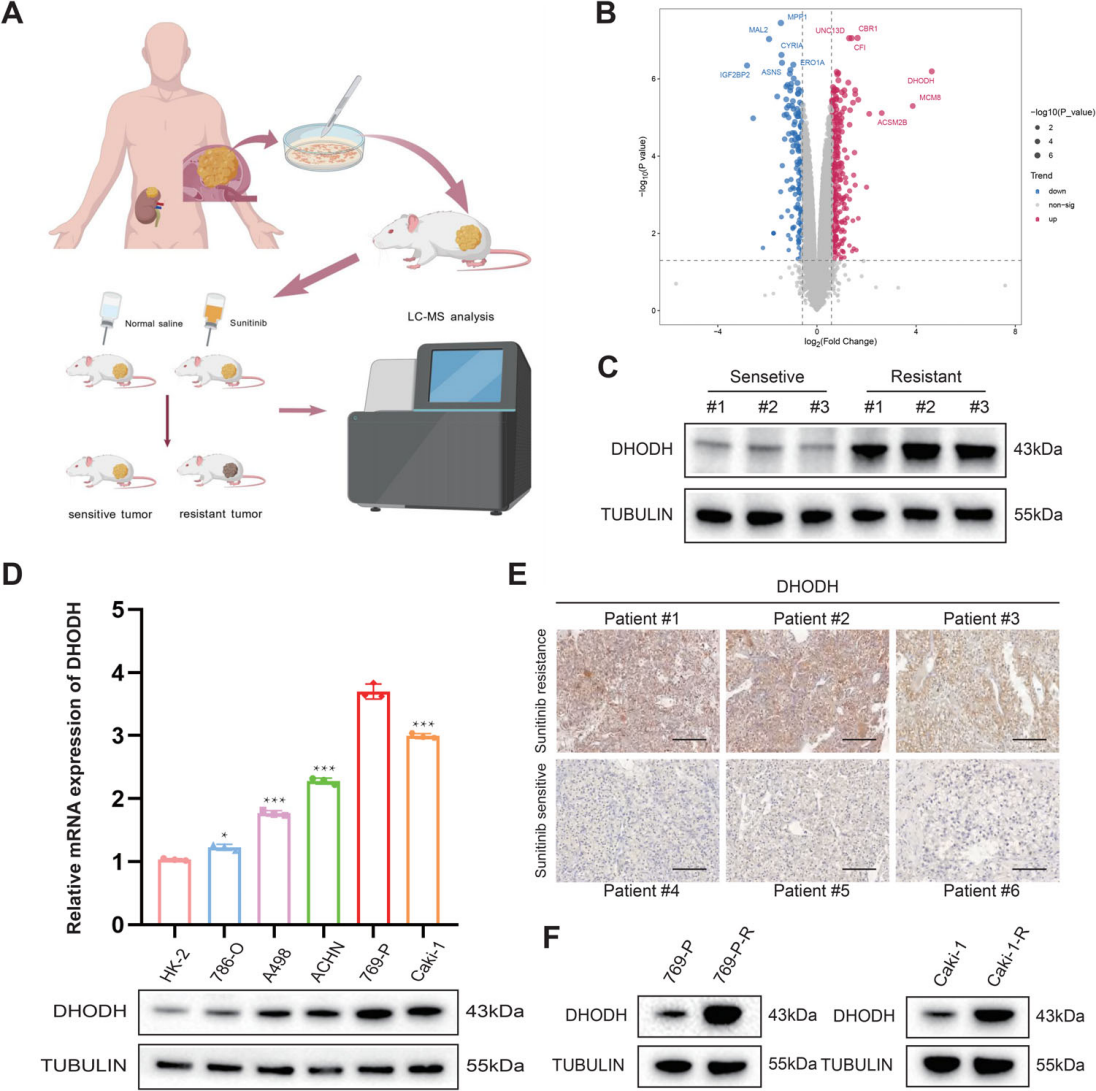
DHODH is highly expressed in sunitinib‐resistant RCC PDX mouse models, patient tissues, and cell lines. (A) Flowchart of RCC patient‐derived xenograft (PDX) model establishment and Treatment. (B) Volcano plot illustrating the differentially expressed genes (DEGs) between sunitinib‐sensitive and sunitinib‐resistant RCC PDX models. (C) Western blotting analysis of DHODH in sunitinib‐sensitive and sunitinib‐resistant RCC patient tissues. (D) Western blotting and QRT‐PCR analyses of DHODH in the HK‐2 cell line and five RCC cell lines. The *p* values were determined by Student's *t*‐test. ^*^ *P* < 0.05, ^**^ *P* < 0.01, ^***^ *P* < 0.001. (E) Pictures of IHC staining for DHODH in three pairs of sunitinib‐resistant and sunitinib‐sensitive RCC tissues. Scale bars, 100 µm. (F) Western blotting analysis of DHODH in 769‐P and Caki‐1 cells with or without sunitinib resistance. The data are presented as the means ± SDs (n = 3). Ns, not significant; ^*^ *P* < 0.05, ^**^ *P* < 0.01, ^***^ *P* < 0.001.

To investigate the mechanisms underlying sunitinib resistance in RCC, we collected tumor tissues from three distinct RCC patients to establish patient‐derived xenograft (PDX) mouse models. These models were then treated with either sunitinib or saline control to establish sunitinib‐sensitive and ‐resistant RCC PDX models. With continuous sunitinib treatment, the sensitivity of tumors in the sunitinib‐treated group to sunitinib gradually decreased. In the third‐generation PDX models, the tumor growth rate in the sunitinib‐treated group was comparable to that in the normal saline group, confirming that the tumors had developed resistance to sunitinib. Subsequently, we performed proteomic sequencing analysis to explore the protein changes associated with the development of sunitinib resistance in RCC (Figure 1A). Proteomic sequencing revealed DHODH as the most significantly upregulated protein among all detected proteins in sunitinib‐resistant tumors (Figure 1B).

To investigate the association between DHODH and sunitinib resistance in RCC, Western blot assays and immunohistochemistry (IHC) staining confirmed that DHODH was more highly expressed in sunitinib‐resistant RCC tumor tissues compared with sunitinib‐sensitive counterparts (Figure 1C,E). We further established sunitinib‐resistant RCC cell lines (769‐P‐R and Caki‐1‐R). Western blot analysis confirmed that DHODH protein levels were elevated in these resistant lines compared to their parental counterparts (Figure 1F). Moreover, DHODH expression was universally elevated in RCC tumor tissues compared to adjacent normal tissues, as demonstrated by Western blot, QRT‐PCR, and IHC staining (Figure S1A–C). Additionally, DHODH is overexpressed in RCC cell lines (786‐O, A498, ACHN, 769‐P and Caki‐1) at the protein and transcript levels compared to normal proximal tubule epithelial cell line HK2 cells. Notably, the upregulation was most significant in the 769‐P and Caki‐1 cell lines (Figure 1D). Accumulating evidence has demonstrated a link between DHODH and sunitinib resistance in RCC.

### 3.2 DHODH Overexpression Promotes Sunitinib Resistance in RCC Both in Vitro and in Vivo

**FIGURE 2:**
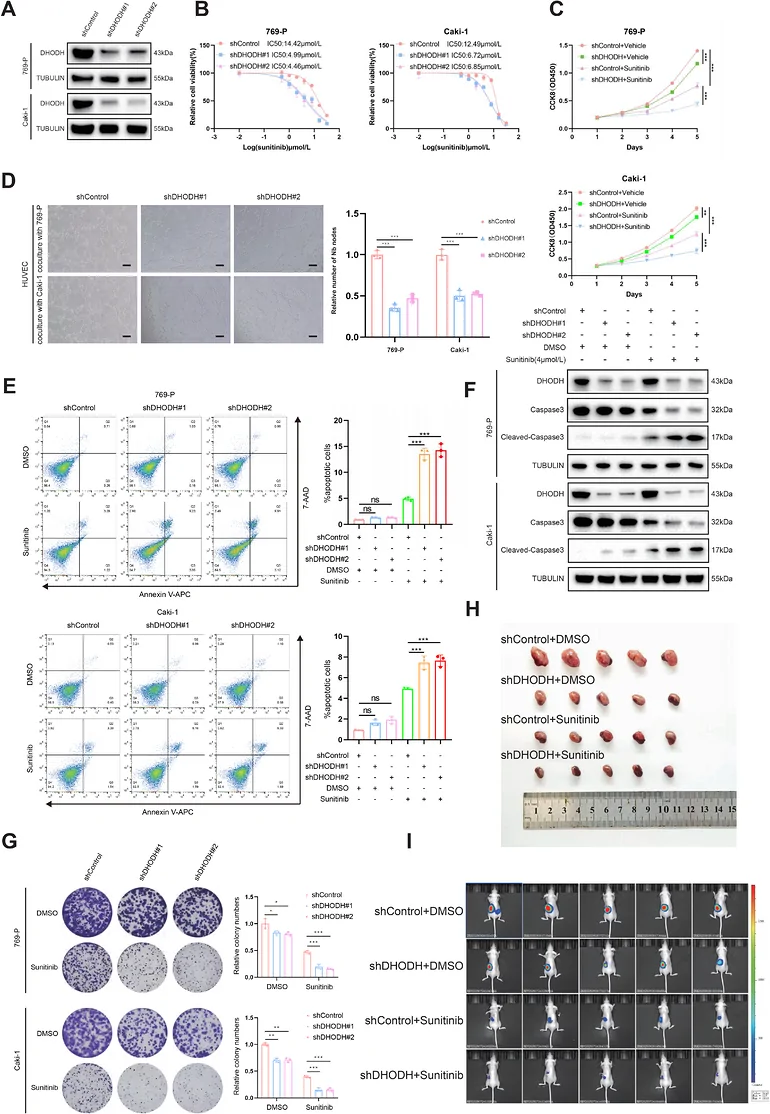
DHODH overexpression promotes sunitinib resistance in RCC both in vitro and in vivo. (A) Western blotting analysis of DHODH in 769‐P and Caki‐1 cells with or without DHODH knockdown (KD). (B) 769‐P and Caki‐1 cells with or without DHODH KD were treated with a range of sunitinib concentrations for 24 h and subjected to CCK‐8 assay. The IC50 values of sunitinib for each group are shown in the figures. (C) 769‐P and Caki‐1 cells with or without DHODH KD were treated with or without sunitinib (2 µm) for 5 days and subjected to CCK‐8 assay. (D) Pictures of In vitro angiogenesis assay at 8 h after cells were plated on Matrigel. HUVECs were co‐cultured at 769‐P and Caki‐1 cells with or without DHODH KD for 24 h. Scale bars, 100 µm. (E‐F) 769‐P and Caki‐1 cells with or without DHODH KD were treated with or without sunitinib (4 µm) for 24 h. Cells were collected for Flow cytometry analysis of apoptosis (E) and Western blot analysis (F). (G) Colony formation assays of 769‐P and Caki‐1 cells with or without DHODH KD, treated with or without sunitinib (2 µm) for two weeks. (H) Picture of xenograft orthotopic RCC model with or without DHODH KD was treated with sunitinib (40 mg/kg) or DMSO via oral gavage once daily for a continuous 4‐week regimen. (I) Bioluminescent images of the formed tumors at the end of the experiments. The data are presented as the means ± SDs (n = 3). For animal studies, each group contained 5 mice (n = 5). Ns, not significant; ^*^ *P* < 0.05, ^**^ *P*< 0.01, ^***^ *P*< 0.001. The *p* values of C, D, E, and G were determined by Student's *t*‐test. ^*^ *P*< 0.05, ^**^ *P*< 0.01, ^***^ *P*< 0.001.

To investigate the functional role of DHODH in sunitinib resistance, we generated 769‐P and Caki‐1 and their drug‐resistant cell lines with stable DHODH KD or overexpression, and the efficiency of DHODH modulation was verified by Western blot assays. (Figure 2A, Figure S2A,G). CCK‐8 assays showed that DHODH KD significantly reduced the half‐maximal inhibitory concentration (IC50) of sunitinib (Figure 2B, Figure S2H). Consistent with this, colony formation assays demonstrated that DHODH KD markedly inhibited cell proliferation after sunitinib treatment (Figure 2C,G; Figure S2I,J). Moreover, DHODH KD enhanced sunitinib‐induced apoptosis (Figure 2E,F). Additionally, inhibiting DHODH expression in 769‐P and Caki‐1 cells reduced their pro‐angiogenic capability, as measured by the HUVEC tubule formation assay.(Figure 2D). Conversely, DHODH overexpression promoted a resistant phenotype: it increased the IC50 of sunitinib, enhanced cell proliferation and pro‐angiogenic capacity, and suppressed sunitinib‐induced apoptosis (Figure S2B–F).

To evaluate the in vivo function of DHODH, we established orthotopic xenograft models in nude mice using 769‐P cells. Four weeks post‐implantation, mice were treated daily by oral gavage with either sunitinib (40 mg/kg) or DMSO for four weeks. Notably, either knockdown of DHODH alone or treatment with sunitinib alone suppressed tumor growth, whereas the combination of DHODH KD and sunitinib administration resulted in a significantly more pronounced inhibition of tumor growth. These findings demonstrate that DHODH KD synergistically enhances the anti‐tumor efficacy of sunitinib in vivo (Figure 2H,I). Additionally, similar phenomena were observed in subcutaneous xenograft models using 769‐P‐R cells (Figure S2K).

### 3.3 DHODH Promotes Sunitinib Resistance in RCC via Its Non‐Enzymatic Activity

**FIGURE 3:**
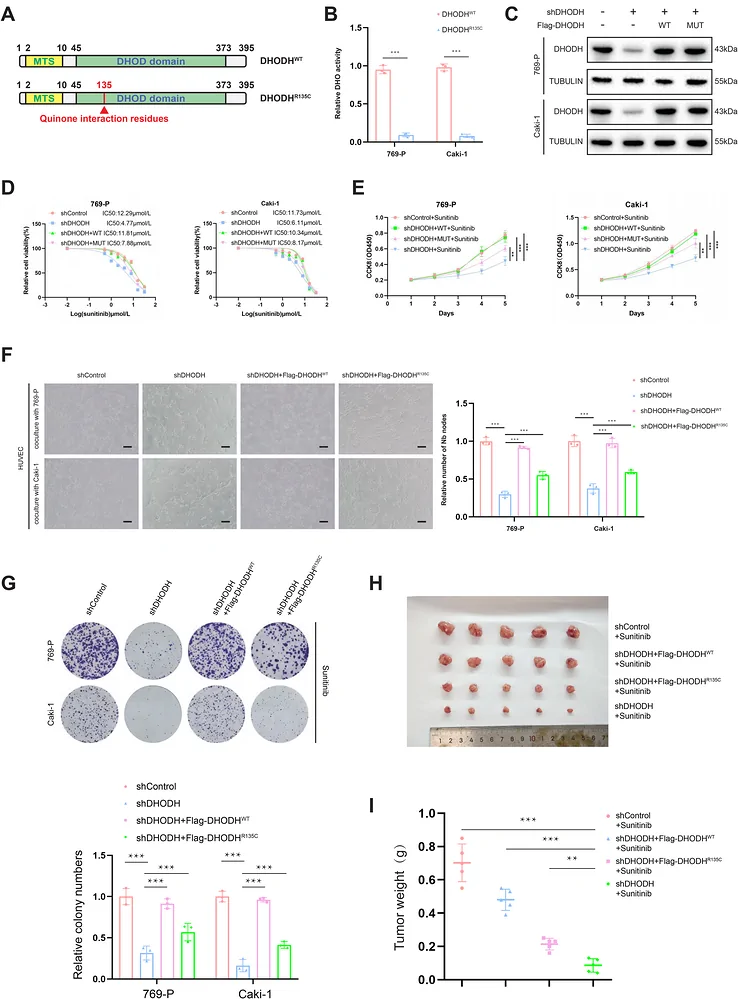
The nonenzymatic function of DHODH is required for sunitinib resistance in RCC. (A) Schematic Representation of the Enzyme‐Dead DHODH Mutant and Its Functional Consequence. (B) DHO activity was measured in cells expressing either wild‐type (DHODH‐WT) or mutant (DHODH‐MUT) enzyme. (C) Western blot analysis of DHODH in DHODH KD RCC cells re‐introducing DHODH‐WT or DHODH‐MUT. (D) DHODH KD RCC cells re‐introducing DHODH‐WT or DHODH‐MUT, were treated with a range of sunitinib concentrations for 24 h and subjected to CCK‐8 assay. The IC50 values of sunitinib for each group are shown in the figures. (E) DHODH KD RCC cells re‐introducing DHODH‐WT or DHODH‐MUT, were treated with or without sunitinib (2 µm) for five days and subjected to CCK‐8 assay. (F) Pictures of In Vitro Angiogenesis Assay at 8 h after cells were plated on Matrigel. HUVECs were co‐cultured at DHODH KD RCC cells re‐introducing DHODH‐WT or DHODH‐MUT for 24 h. Scale bars, 100 µm. (G) Colony formation assays of DHODH KD RCC cells re‐introducing DHODH‐WT or DHODH‐MUT, treated with or without sunitinib (2 µm) for two weeks. (H) Picture of subcutaneous xenografts. (I) Final tumor weights. The data are presented as the means ± SDs (n = 3). For animal studies, each group contained 5 mice (n = 5). Ns, not significant; ^*^ *P* < 0.05, ^**^ *P* < 0.01, ^***^ *P* < 0.001. The *p* values of E, F, G, and I were determined by Student's *t*‐test. Ns, not significant; ^*^ *P* < 0.05, ^**^ *P* < 0.01, ^***^ *P* < 0.001.

Dihydroorotate dehydrogenase (DHODH) is a rate‐limiting enzyme in the de novo pyrimidine biosynthesis pathway, which sustains the pyrimidine supply required for cell proliferation by catalyzing the oxidation of dihydroorotate to orotate. To determine whether DHODH promotes sunitinib resistance in RCC via its enzymatic activity, we generated an enzymatically inactive DHODH mutant (R135C, DHODH‐MUT), and its ability to abrogate DHODH enzymatic activity was verified (Figure 3A,B) [20]. Wild‐type DHODH (DHODH‐WT) or DHODH‐MUT was reconstituted into DHODH KD cells at levels approximately equivalent to the physiological expression level (Figure 3C). Consistent with the findings in Figure 2B–D, G, DHODH KD significantly reduced the IC50 and tube formation capacity of RCC cells, and suppressed cell proliferation following sunitinib treatment. Intriguingly, reconstitution of DHODH‐WT completely reversed these inhibitory effects, whereas reintroduction of DHODH‐MUT only partially, but significantly, reversed such suppression (Figure 3D–G). Additionally, similar phenomena were observed in vitro experiments (Figure 3H,I). Furthermore, we found that exogenous uridine supplementation only partially reverses sunitinib resistance in RCC cells (Figure S3A–C). Collectively, these findings demonstrate that, beyond its enzymatic activity, DHODH promotes sunitinib resistance in RCC through an independent non‐enzymatic function.

### 3.4 DHODH Regulates the Transcription of VEGFA Through TRIM28

**FIGURE 4:**
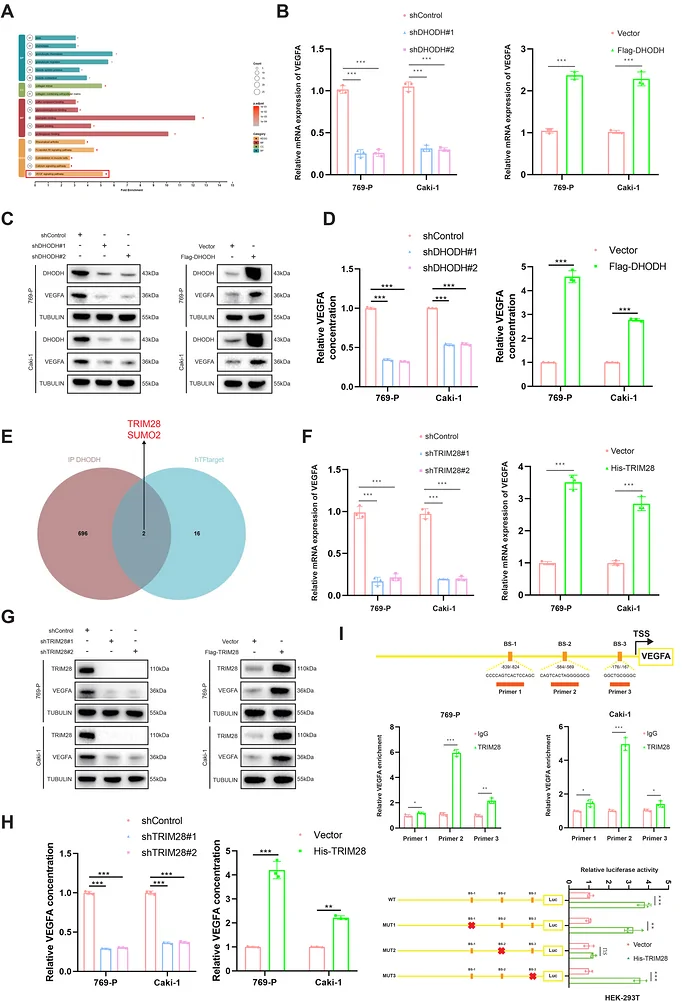
DHODH promotes TRIM28‐mediated transcriptional activation of VEGFA. (A) GO and KEGG enrichment analysis. (B) VEGFA transcript levels were quantified by QRT‐PCR in 769‐P and Caki‐1 cells with DHODH KD or overexpression. (C) VEGFA protein levels were analyzed by Western blot in 769‐P and Caki‐1 cells with DHODH KD or overexpression. (D) VEGFA secretion was measured by ELISA in the conditioned medium from 769‐P and Caki‐1 cells with DHODH KD or overexpression. (E) Venn diagram identifying candidate genes at the intersection between DHODH‐bound genes (from Flag‐IP) and transcription factors of TRIM28 predicted by the hTFtarget database. (F) VEGFA transcript levels were quantified by QRT‐PCR in 769‐P and Caki‐1 cells with TRIM28 KD or overexpression. (G) VEGFA protein levels were analyzed by Western blot in 769‐P and Caki‐1 cells with TRIM28 KD or overexpression. (H) VEGFA secretion was measured by ELISA in the conditioned medium from 769‐P and Caki‐1 cells with TRIM28 KD or overexpression. (I) To validate TRIM28 binding, the VEGFA promoter was analyzed in three fragments spanning predicted binding sites (BS), followed by primer design and CUT&RUN assay. To functionally validate the predicted TRIM28 binding sites (BS‐1, BS‐2, and BS‐3), we generated a series of luciferase reporter plasmids containing truncations or site‐directed mutations within the VEGFA promoter. The binding activity of TRIM28 was then assessed by dual‐luciferase reporter assays in 293T cells following co‐transfection of these reporter constructs with a Renilla internal control plasmid. The data are presented as the means±SDs (n = 3). Ns, not significant; ^*^ *P* < 0.05, ^**^ *P* < 0.01, ^***^ *P* < 0.001. The *p* values of B, D, F, H, and I were determined by Student's *t*‐test. Ns, not significant; ^*^ *P* < 0.05, ^**^ *P* < 0.01, ^***^ *P* < 0.001.

To further explore the downstream mechanisms underlying DHODH‐mediated sunitinib resistance in RCC, we performed RNA sequencing (RNA‐seq) in DHODH KD and control 769‐P cells. Notably, Kyoto Encyclopedia of Genes and Genomes (KEGG) pathway enrichment analysis revealed that DHODH activated the VEGF signaling pathway, which exhibited the highest fold enrichment (Figure 4A). Extensive studies have established that sunitinib exerts its anti‐tumor efficacy by inhibiting vascular endothelial growth factor receptors (VEGFRs) and platelet‐derived growth factor receptors (PDGFRs), which in turn abrogates tumor angiogenesis and restricts cell proliferation, ultimately leading to the effective control of tumor progression [11, 21]. Thus, RNA‐seq analysis suggests that DHODH may promote sunitinib resistance in RCC by activating the VEGF signaling pathway. Meanwhile, as the most critical factor in the VEGF signaling pathway, vascular endothelial growth factor A (VEGFA) serves as a core signaling protein regulating tumor angiogenesis and vascular permeability, thereby contributing to sunitinib resistance in RCC. Consequently, we focused our subsequent studies on VEGFA.

Initially, we found that VEGFA mRNA, protein, and supernatant levels were correspondingly decreased following DHODH KD or increased upon DHODH overexpression (Figure 4B–D). Since DHODH modulated VEGFA expression at the transcriptional level, we systematically integrated data from multiple sources to identify potential transcription factors. Cross‐analysis of two datasets—including proteins that interact with DHODH detected by immunoprecipitation‐based mass spectrometry (IP‐MS) and transcription factors from the hTFtarget database‐yielded two overlapping candidates: TRIM28 and SUMO2 (Figure 4E). Subsequently, we found that TRIM28 KD or overexpression affected VEGFA expression (Figure 4F–H), whereas SUMO2 had no such effect (Figure S4A–C). We predicted three potential TRIM28 binding sites within the VEGFA promoter using the hTFtarget database (Figure S4D), and CUT & RUN assays further confirmed that TRIM28 is mainly recruited to the BS‐2 site. To further validate these findings, we constructed truncated reporter plasmids for each binding site. Dual‐luciferase reporter assays demonstrated that mutation of the BS‐2 site significantly inhibited TRIM28‐mediated transcriptional activation of VEGFA, indicating that the BS‐2 site is responsible for TRIM28‐dependent transcriptional regulation of VEGFA (Figure 4I). Furthermore, we found that TRIM28 does not affect the ubiquitination level of VEGFA, nor does it alter the histone modification status at the VEGFA promoter region (Figure S4E,F). Collectively, these findings validate that TRIM28 directly binds to the promoter region of VEGFA to induce its transcriptional activation.

### 3.5 DHODH Interacts with TRIM28 and Inhibits Its Ubiquitination

**FIGURE 5:**
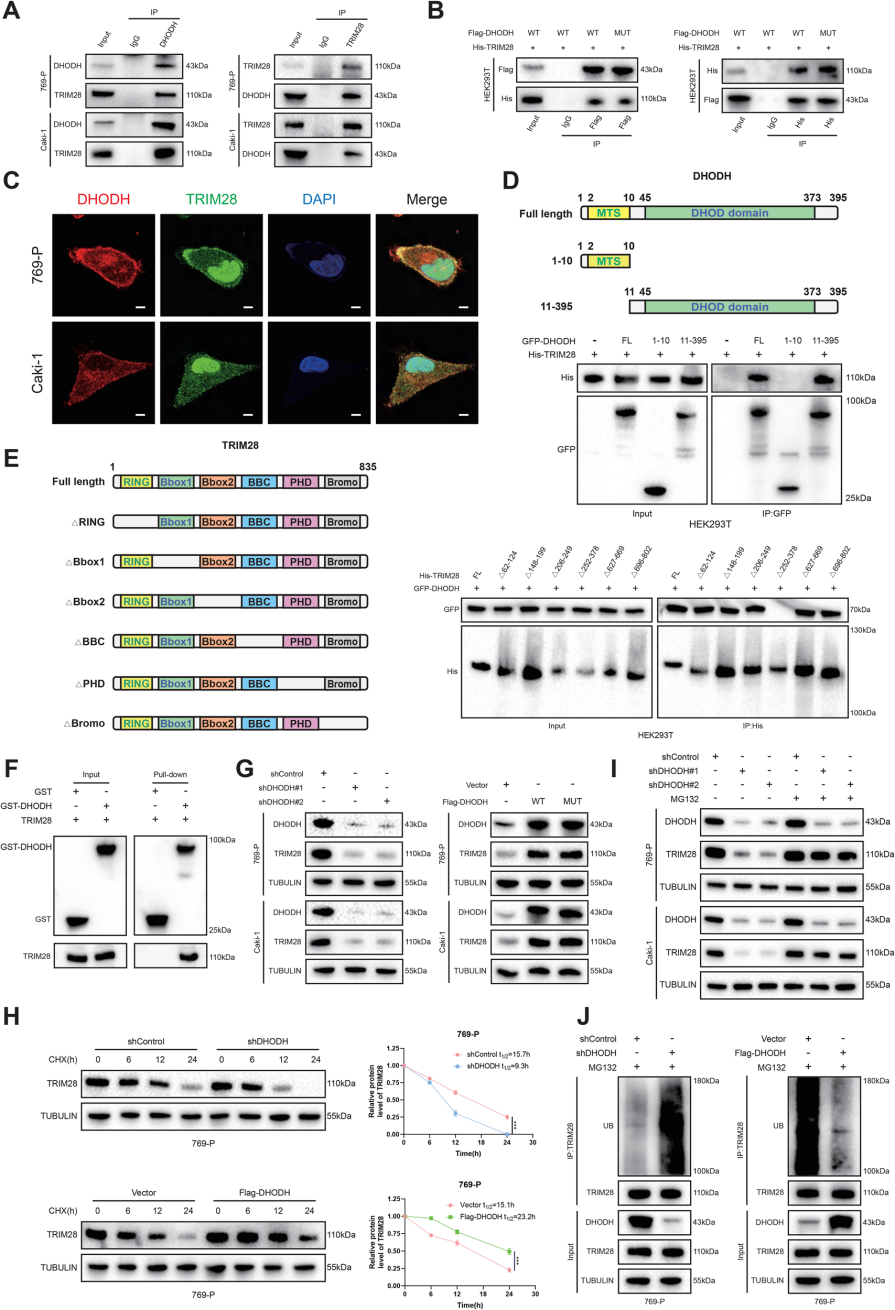
DHODH interacts with TRIM28 and stabilizes it by inhibiting its ubiquitination. (A) The endogenous interaction between DHODH and TRIM28 was confirmed by reciprocal co‐immunoprecipitation (Co‐IP) in 769‐P and Caki‐1 cells. (B) Co‐IP analysis comparing the interaction of His‐TRIM28 with either DHODH‐WT or DHODH‐MUT. (C) Representative immunofluorescence confocal images show co‐localization of DHODH and TRIM28 in 769‐P and Caki‐1 cells. Scale bar, 90 µm. (D) Schematic Diagram of DHODH Truncation Mutant Design. To map the TRIM28‐interacting domain of DHODH, HEK293T cells were co‐transfected with His‐TRIM28 and either full‐length or truncated GFP‐DHODH constructs. The DHODH complexes were immunoprecipitated using GFP‐Trap beads and analyzed by immunoblotting with an anti‐His antibody. (E) Schematic Diagram of TRIM28 Truncation Mutant Design. To map the DHODH‐interacting domain of TRIM28, HEK293T cells were co‐transfected with GFP‐DHODH and either full‐length or truncated His‐TRIM28 constructs. The TRIM28 complexes were immunoprecipitated using His‐Trap beads and analyzed by immunoblotting with an anti‐GFP antibody. (F) Western blot analysis of TRIM28 GST‐pulldown by GST‐DHODH recombinant. (G) TRIM28 protein levels were analyzed by Western blot in 769‐P and Caki‐1 cells with DHODH KD or overexpressing DHODH‐WT or DHODH‐MUT. (H) Western blot analysis of TRIM28 in DHODH KD, overexpression, and corresponding control 769‐P cells following treatment with cycloheximide (CHX) for the indicated times. Band intensities were quantified and plotted (right). (I) Western blot analysis of TRIM28 in DHODH KD 769‐P and Caki‐1 cells treated with or without MG132. (J) Following transfection with shControl, shDHODH, or Flag‐DHODH, 769‐P cells were treated with the proteasome inhibitor MG132 for 10 h. TRIM28 was then immunoprecipitated from cell lysates and subjected to immunoblotting analysis to detect co‐precipitated ubiquitin. The data are presented as the means ± SDs (n = 3). Ns, not significant; ^*^ *P* < 0.05, ^**^ *P* < 0.01, ^***^ *P* < 0.001. The *p* values of G were determined by Student's *t*‐test. Ns, not significant; ^*^ *P* < 0.05, ^**^ *P* < 0.01, ^***^ *P* < 0.001.

To investigate the regulatory effect of DHODH on TRIM28, we first confirmed via Co‐IP and immunofluorescence assays that endogenously expressed DHODH and TRIM28 interacted with each other in 769‐P and Caki‐1 cells. Additionally, ectopically overexpressed DHODH and TRIM28 also bound to each other in 293T cells, and this interaction was independent of the enzymatic activity of DHODH (Figure 5A–C). To identify the specific domains responsible for the physical interaction between DHODH and TRIM28, we further constructed a series of truncated plasmids of DHODH and TRIM28. Subsequent analyses revealed that both the DHOD domain of DHODH and the BBC domain of TRIM28 are indispensable for the physical interaction between these two proteins (Figure 5D,E). GST pull‐down assays also confirmed that DHODH physically interacts with TRIM28 (Figure 5F).

Notably, DHODH KD or overexpression had no effect on TRIM28 mRNA expression, yet it led to a concomitant reduction or elevation in TRIM28 protein levels, and this regulatory effect was independent of the enzymatic activity of DHODH (Figure 5G, S5A). This indicates that DHODH likely modulates TRIM28 protein stability through a post‐transcriptional regulatory mechanism. Subsequently, upon treatment with cycloheximide (CHX), a protein synthesis inhibitor, we observed that DHODH KD significantly shortened the half‐life of TRIM28 (Figure 5H). In contrast, overexpression of DHODH significantly prolonged the half‐life of TRIM28 protein (Figure S4B). Considering that the ubiquitin‐proteasome pathway and autophagy‐lysosome pathway are two key pathways that regulate protein degradation at the post‐transcriptional level, we treated 769‐P and Caki‐1 cells with the proteasome inhibitor MG‐132 and the lysosome inhibitor CQ to clarify which pathway mediates TRIM28 degradation. As shown by the results, treatment with MG132 rescued the DHODH KD‐driven decrease in TRIM28 protein levels, but CQ failed to exert a rescue effect (Figure 5I; Figure S4C). Moreover, we found that DHODH KD significantly increased TRIM28 ubiquitination, while overexpression of DHODH produced the opposite outcome (Figure 5J; Figure S4D). Taken together, these experimental results validate that DHODH directly binds to TRIM28 and inhibits its ubiquitination. In addition, we found that TRIM28 has no regulatory effect on DHODH protein expression (Figure S5E).

### 3.6 DHODH Inhibits TRIM37‐Mediated Ubiquitination of TRIM28 at Lysine 366 to Suppress Its Proteasomal Degradation

**FIGURE 6:**
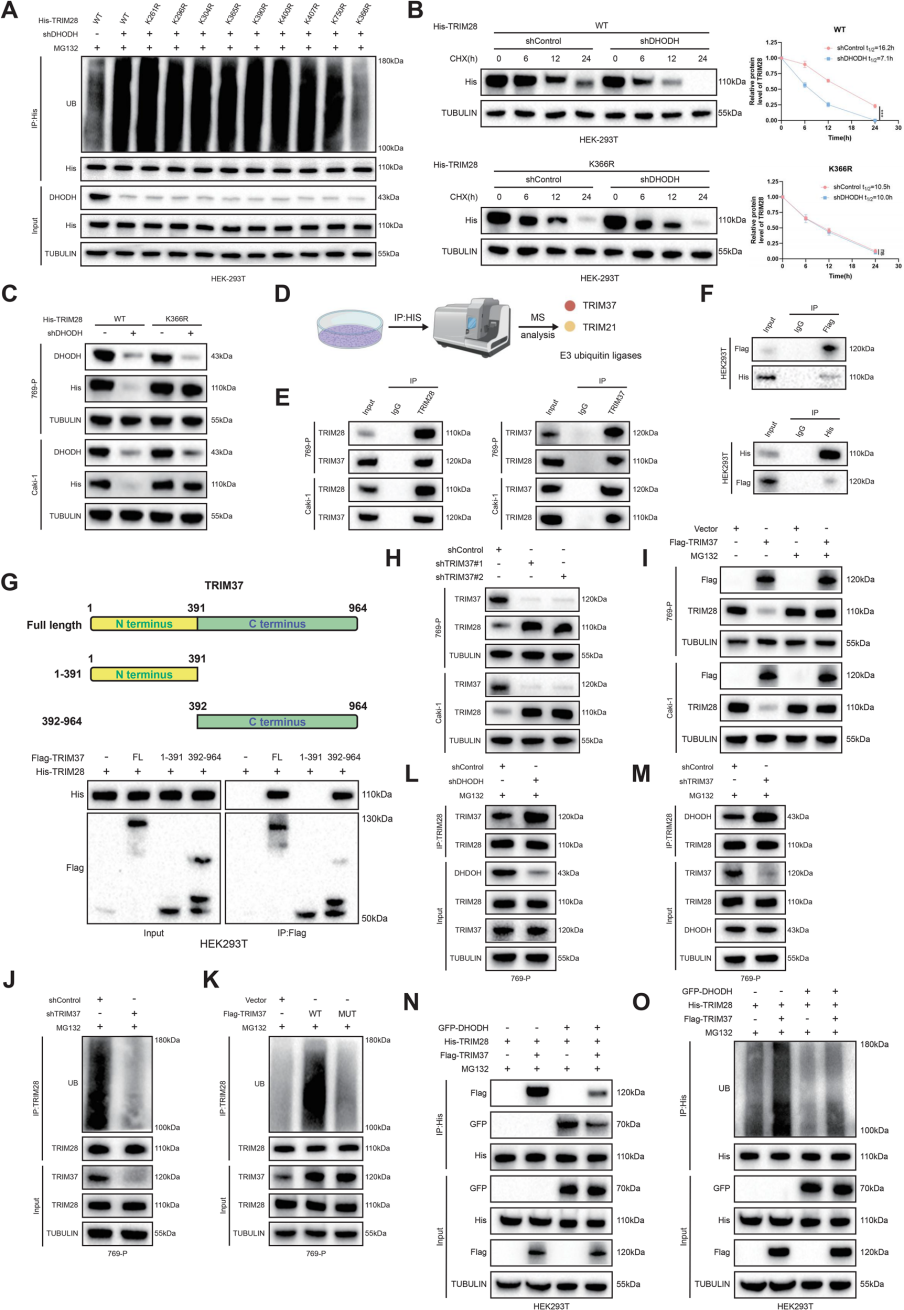
DHODH Inhibits TRIM37‐Mediated TRIM28 Ubiquitination at Lysine 366 to Prevent TRIM28 Proteasomal Degradation. (A) The ubiquitination status of wild‐type and a series of lysine‐to‐arginine (KR) mutant His‐TRIM28 constructs was compared in 293T cells with or without DHODH KD. After MG132 treatment, proteins were immunoprecipitated with an anti‐His antibody and analyzed by Western blotting for ubiquitin modification. (B) The stability of wild‐type and K366R mutant His‐TRIM28 was analyzed in 293T cells with or without DHODH KD. Following treatment with cycloheximide (CHX) to block new protein synthesis, lysates were harvested at the indicated times and subjected to immunoblotting. Band intensities were quantified and plotted (right). (C) We expressed His‐TRIM28 (WT or K366R mutant) in 769‐P and Caki‐1 cells with or without DHODH KD. TRIM28 levels were then detected by immunoblotting with indicated antibodies. (D) Workflow for identifying the E3 ubiquitin ligase responsible for TRIM28 ubiquitination. (E) The endogenous interaction between TRIM37 and TRIM28 was confirmed by reciprocal Co‐IP in 769‐P and Caki‐1 cells. (F) Reciprocal Co‐IP confirms the interaction between ectopically expressed TRIM37 and TRIM28. (G) Schematic Diagram of TRIM37 Truncation Mutant Design. To map the TRIM28‐interacting domain of TRIM37, HEK293T cells were co‐transfected with His‐TRIM28 and either full‐length or truncated Flag‐TRIM37 constructs. The TRIM37 complexes were immunoprecipitated using Flag‐Trap beads and analyzed by immunoblotting with an anti‐His antibody. (H) TRIM28 protein levels were analyzed by Western blot in 769‐P and Caki‐1 cells with TRIM37 KD. (I) Western blot analysis of TRIM28 in TRIM37 overexpression 769‐P and Caki‐1 cells treated with or without MG132. (J‐K) Following transfection with shControl, shTRIM37, TRIM37‐WT, or TRIM37‐MUT, 769‐P cells were treated with the proteasome inhibitor MG132 for 10 h. TRIM28 was then immunoprecipitated from cell lysates and subjected to immunoblotting analysis to detect co‐precipitated ubiquitin. (L‐M) Following transfection with shDHODH (L) or shTRIM37 (M), 769‐P cells were treated with the proteasome inhibitor MG132 for 10 h. TRIM28 was then immunoprecipitated from cell lysates and subjected to immunoblotting analysis with the indicated antibodies. (N‐O) Following transfection with His‐TRIM28, GFP‐DHODH, and/or Flag‐TRIM37, 293T cells were treated with the proteasome inhibitor MG132 for 10 h. TRIM28 was then immunoprecipitated from cell lysates and subjected to immunoblotting analysis with the indicated antibodies. The data are presented as the means ± SDs (n = 3). Ns, not significant; ^*^ *P* < 0.05, ^**^ *P* < 0.01, ^***^ *P* < 0.001. The *p* values of B were determined by Student's *t*‐test. Ns, not significant; ^*^ *P* < 0.05, ^**^ *P* < 0.01, ^***^ *P* < 0.001.

To precisely identify the specific ubiquitination sites of TRIM28, we performed ubiquitomic analysis. As a result, nine lysine residues (K261, K296, K304, K365, K366, K390, K400, K407 and K750) were identified as potential ubiquitination sites within TRIM28. Subsequently, we constructed His‐TRIM28 mutant plasmids by replacing each of these lysine residues with arginine, resulting in nine mutants (K261R, K296R, K304R, K365R, K366R, K390R, K400R, K407R, and K750R), respectively. Western blot assays revealed that DHODH KD significantly enhanced the ubiquitination level of TRIM28. Importantly, among all the constructed mutants, only the K366R mutant, but not the other eight mutants, significantly suppressed this enhancement, and it also rescued the downregulation of TRIM28 protein levels caused by DHODH knockdown (Figure 6A,C). In addition, DHODH KD significantly shortened the half‐life of wild‐type TRIM28 protein, while the half‐life of the K366R mutant remained unchanged (Figure 6B).

We then performed mass spectrometry following immunoprecipitation to identify the potential E3 ubiquitin ligase required for TRIM28 ubiquitination. As presented in Figure 6D, we successfully identified two potential E3 ubiquitin ligases that mediate TRIM28 ubiquitination: TRIM37 and TRIM21. Notably, co‐ip and immunofluorescence showed that endogenously expressed TRIM37 and TRIM28 interacted with each other in 769‐P and Caki‐1 cells, and ectopically overexpressed TRIM37 and TRIM28 also bound with each other in 293T cells (Figure 6E,F; Figure S6B). In contrast, no interaction between TRIM21 and TRIM28 was observed under the same experimental conditions (Figure S6A). To identify the specific domains responsible for the physical interaction between TRIM37 and TRIM28, we further constructed a series of truncated plasmids for TRIM37. Notably, the results demonstrated that TRIM28 binds to the C‐terminus of TRIM37 via its own BBC domain (Figure 6G; Figure S6C), which is identical to the domain bound by DHODH. GST pull‐down assays also confirmed that TRIM37 physically interacts with TRIM28 (Figure 6D).

To confirm whether TRIM37 serves as the E3 ubiquitin ligase that mediates TRIM28 ubiquitination and subsequent degradation, we found that TRIM37 KD leads to an increase in TRIM28 protein levels, whereas TRIM37 overexpression resulted in a reduction in TRIM28 protein levels. Meanwhile, we constructed an enzymatically inactive mutant of TRIM37 (C18R, TRIM37‐MUT) [22]. Intriguingly, we observed that transfection with the enzymatically inactive TRIM37‐MUT failed to decrease TRIM28 protein levels (Figure 6H; Figure S6G). Subsequently, we treated RCC cells with MG132, and we found that MG132 treatment abolished the TRIM28 reduction induced by TRIM37 overexpression (Figure 6I). Meanwhile, after treatment with CHX, we observed that TRIM37 overexpression significantly shortened the half‐life of TRIM28, whereas TRIM37 KD markedly prolonged it (Figure S6E,F). We further found that overexpression of TRIM37 prominently increased TRIM28 ubiquitination, whereas TRIM37 KD decreased the ubiquitination level of TRIM28 protein. Notably, the TRIM37‐MUT had no effect on TRIM28 ubiquitination (Figure 6J‐K; Figure S6H,I). Intriguingly, our results showed that DHODH KD significantly enhanced the interaction between TRIM37 and TRIM28, and conversely, TRIM37 KD also markedly increased the interaction between DHODH and TRIM28 (Figure 6L,M; Figure S6J,K). In addition, we found that DHODH overexpression inhibited the interaction between TRIM28 and TRIM37 and suppressed TRIM28 ubiquitination. Similarly, TRIM37 overexpression repressed the interaction between TRIM28 and DHODH and promoted TRIM28 ubiquitination (Figure 6N,O). Collectively, these results demonstrate that DHODH competes with TRIM37 for binding to TRIM28, thereby inhibiting TRIM37‐mediated ubiquitination and degradation of TRIM28.

### 3.7 DHODH Promotes Sunitinib Resistance in RCC by Upregulating TRIM28

**FIGURE 7:**
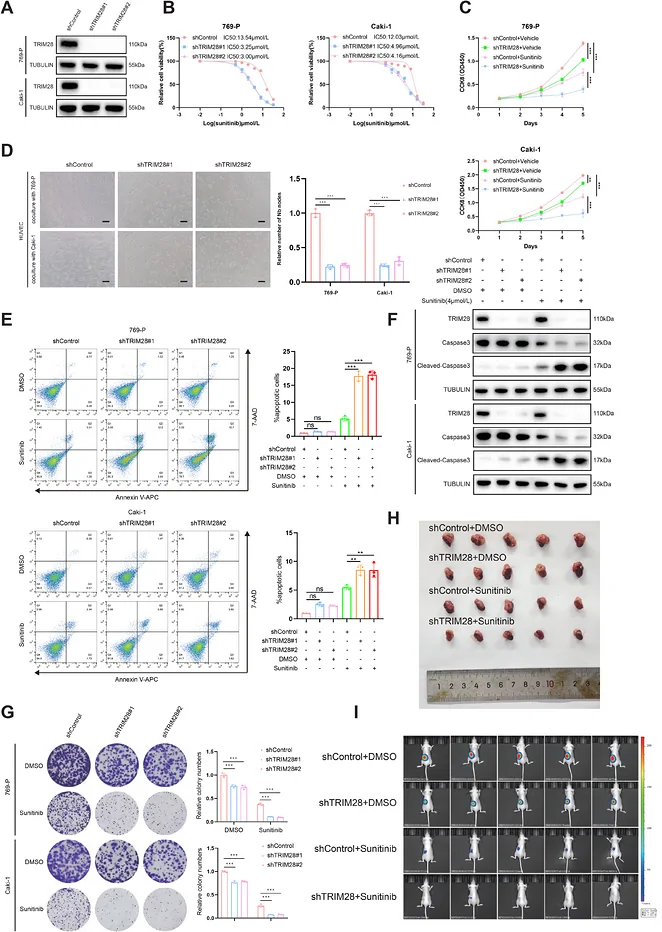
Overexpression of TRIM28 drives sunitinib resistance in RCC both in vitro and in vivo. (A) Western Blot Analysis Validating TRIM28 KD in 769‐P and Caki‐1 cells. (B) 769‐P and Caki‐1 cells with or without TRIM28 KD were treated with a range of sunitinib concentrations for 24 h and subjected to CCK‐8 assay. The IC50 values of sunitinib for each group are shown in the figures. (C) 769‐P and Caki‐1 cells with or without TRIM28 KD were treated with or without sunitinib (2 µm) for five days and subjected to CCK‐8 assay. (D) Pictures of the In Vitro Angiogenesis Assay at 8 h after cells were plated on Matrigel. HUVECs were co‐cultured at 769‐P and Caki‐1 cells with or without TRIM28 KD for 24 h. Scale bars, 100 µm. (E‐F) 769‐P and Caki‐1 cells with or without TRIM28 KD were treated with or without sunitinib (4 µm) for 24 h. Cells were collected for Flow cytometry analysis of apoptosis (E) and Western blot analysis (F). (G) Colony formation assays of 769‐P and Caki‐1 cells with or without TRIM28 KD, treated with or without sunitinib (2 µm) for two weeks. (H) Picture of xenograft orthotopic RCC model with or without TRIM28 KD, were treated with sunitinib (40 mg/kg) or DMSO via oral gavage once daily for a continuous 4‐week regimen. (I) Bioluminescent images of the formed tumors at the end of the experiments. The data are presented as the means ± SDs (n = 3). For animal studies, each group contained 5 mice (n = 5). Ns, not significant; ^*^ *P* < 0.05, ^**^ *P* < 0.01, ^***^ *P* < 0.001. The *p* values of C, D, E, and G were determined by Student's *t*‐test. ^*^ *P* < 0.05, ^**^ *P* < 0.01, ^***^ *P* < 0.001.

Given that DHODH mediates TRIM28 upregulation through its non‐enzymatic activity, we further explored the function of TRIM28 in sunitinib resistance in RCC. TRIM28 KD and overexpression were conducted in 769‐P and Caki‐1 cells, and the transfection efficiencies were verified by Western blot (Figure 7A; Figure S6A). Subsequently, CCK‐8 and colony formation assay revealed that TRIM28 KD significantly reduced the IC50 of 769‐P and Caki‐1 cells, and notably inhibited their proliferation after sunitinib exposure (Figure 7B,C, and G). Additionally, TRIM28 KD further promoted apoptosis of these cells treated with sunitinib (Figure 7E,F). Moreover, TRIM28 KD reduced the pro‐angiogenic capability of RCC cell lines, as measured by the HUVEC tubule formation assay (Figure 7D). Conversely, TRIM28 overexpression showed the opposite phenotypes (Figure S6B–F). Additionally, in vivo experiments showed that combination treatment with sunitinib and TRIM28 KD significantly inhibited tumor growth. This result demonstrated that TRIM28 KD synergistically enhanced the antitumor activity of sunitinib in vivo, consistent with our in vitro findings (Figure 7H,I).

To further verify whether TRIM28 acts as a downstream effector of DHODH in regulating sunitinib resistance in RCC, we performed rescue assays by overexpressing TRIM28 in DHODH KD 769‐P and Caki‐1 cells. We found that overexpression of TRIM28 significantly reversed the reduction of sunitinib resistance in RCC cells mediated by DHODH KD (Figure S8A–D). In addition, similar results were also observed in rescue assays by knocking down TRIM28 in DHODH‐overexpressing 769‐P and Caki‐1 cells (Figure S8G–J). Furthermore, we also found the regulation of VEGFA by DHODH is mediated through TRIM28 (Figure S8F). These experimental results confirm that DHODH enhances sunitinib resistance in RCC by upregulating TRIM28 expression.

### 3.8 Lisaftoclax Sensitizes Sunitinib by Preventing the Interaction between DHODH and TRIM28

**FIGURE 8:**
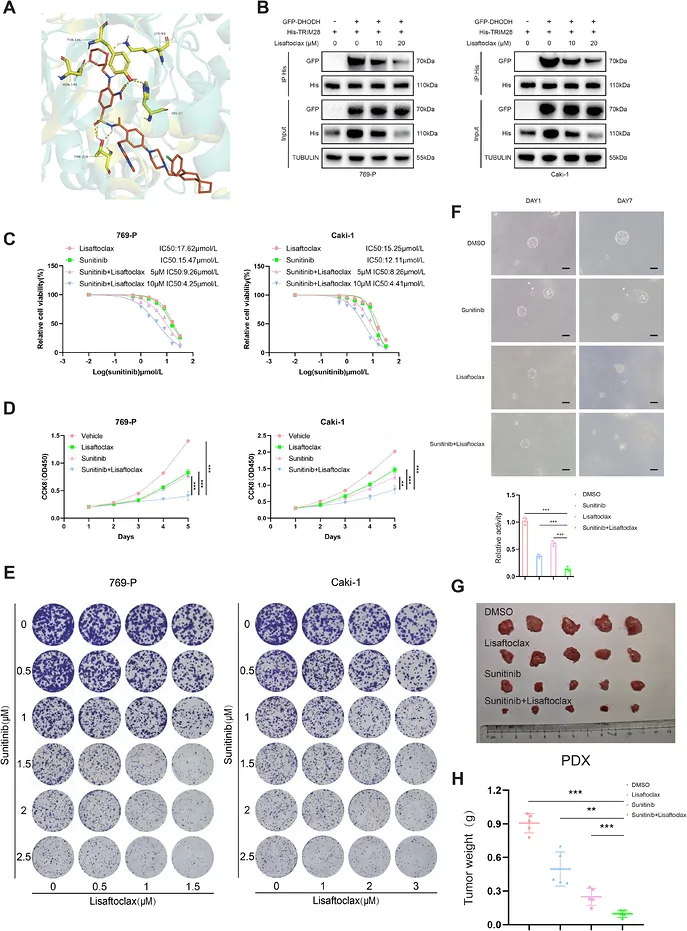
Lisaftoclax Sensitizes Sunitinib by Preventing the Interaction Between DHODH and TRIM28. (A) Schematic of Molecular Docking between Lisaftoclax and DHODH. (B) 769‐P and Caki‐1 cells co‐expressing GFP‐DHODH and His‐TRIM28 were treated with different concentrations of Lisaftoclax for 48 h. Subsequently, His‐TRIM28 was immunoprecipitated from cell lysates and subjected to immunoblotting analysis with the indicated antibodies. (C) 769‐P and Caki‐1 cells were treated with a range of sunitinib or lisaftoclax concentrations alone or the combination of sunitinib plus lisaftoclax (5 or 10 µm) for 24 h and subjected to CCK‐8 assay. The IC50 values of sunitinib for each group are shown in the figures. (D) Cells were treated for five days under four conditions: vehicle control, sunitinib (2 µm) alone, Lisaftoclax (10 µm) alone, or the combination of both drugs and subjected to CCK‐8 assay. (E) Colony formation assays of 769‐P and Caki‐1 cells after co‐treatment with Lisaftoclax and sunitinib indicated. (F) Representative images of RCC PDOs following seven‐day treatment with 10 µm Lisaftoclax and 2 µm sunitinib. Scale bar, 100 µm. (G) Representative images and comparison of tumor sizes in the indicated groups. (H) Final tumor weights. The data are presented as the means ± SDs (n = 3). For animal studies, each group contained 5 mice (n = 5). Ns, not significant; ^*^ *P* < 0.05, ^**^ *P* < 0.01, ^***^ *P* < 0.001. The *p* values of D were determined by Student's *t*‐test. ^*^ *P* < 0.05, ^**^ *P* < 0.01, ^***^ *P* < 0.001.

**FIGURE 9:**
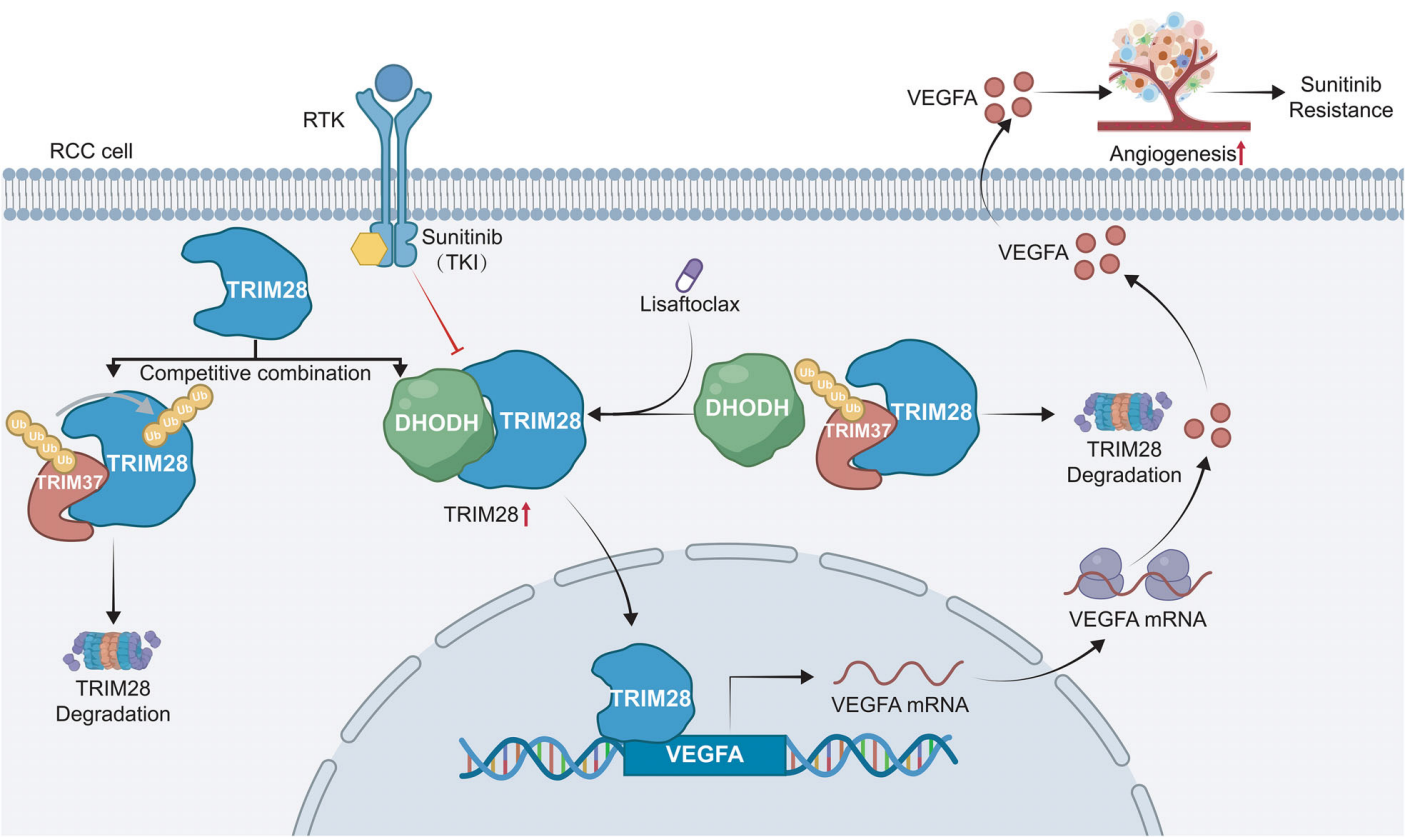
Mechanism of DHODH‐mediated sunitinib resistance via TRIM28 stabilization in RCC. DHODH competes with the E3 ligase TRIM37 for binding to TRIM28, blocking TRIM37‐mediated ubiquitination and degradation of TRIM28. Stabilized TRIM28 activates VEGFA transcription, reactivating the VEGF pathway and conferring sunitinib resistance. Lisaftoclax disrupts the DHODH–TRIM28 interaction, restoring TRIM28 degradation and sunitinib sensitivity.

Given the critical role of DHODH in sunitinib resistance in RCC, we performed a small‐molecule drug screen to target the binding domain between DHODH and TRIM28. Among all screened candidates, Lisaftoclax was the top‐scoring small‐molecule inhibitor, which is predicted to interact with DHODH (affinity (kcal mol^−^^1^) = −18.4) (Figure 8A). Co‐IP experiments after Lisaftoclax treatment in 769‐P and Caki‐1 cells show a dose‐dependent decrease in the binding of GFP‐DHODH to HIS‐TRIM28 (Figure 8B). In addition, lisaftoclax had no impact on DHODH expression as well as its classical enzymatic activity (Figure S8K,L). To further validate the functional effect of lisaftoclax, we found that treatment with lisaftoclax significantly enhanced the sensitivity of 769‐P and Caki‐1 cells to sunitinib (Figure 8C). CCK‐8 and Colony formation assays demonstrated that the combination of sunitinib and Lisaftoclax effectively inhibited cell growth (Figure 8D,E). Moreover, we utilized a patient‐derived organoid (PDO) model to further investigate the function of Lisaftoclax. The combination treatment of sunitinib and Lisaftoclax significantly inhibited the growth of PDOs (Figure 8F). Finally, our results showed that the tumor growth rate was significantly reduced in PDX mice treated with sunitinib combined with lisaftoclax, compared to the sunitinib monotherapy group (Figure 8G,H). These experiments demonstrate that lisaftoclax sensitizes sunitinib in both in vitro and in vivo models. Taken together, these findings demonstrate that the non‐enzymatic activity of DHODH is essential for driving sunitinib resistance in RCC (Figure 9).

## 4 Discussion

The combined use of DHODH inhibitors with other anticancer agents represents a promising strategy for achieving clinical benefit in multiple tumor types. For instance, in pancreatic cancer, leflunomide has been shown to delay the development of gemcitabine resistance by inhibiting DHODH‐mediated de novo pyrimidine synthesis [23]. Intriguingly, in the context of sunitinib‐resistant renal cell carcinoma, we found that mutating the enzymatically active site of DHODH failed to reverse resistance. This suggests that conventional inhibitors targeting the catalytic site may not overcome sunitinib resistance when used in combination. Therefore, we predicted novel drug candidates aimed at an alternative binding site, offering a potential therapeutic strategy for patients with DHODH‐mediated sunitinib resistance.

The DHODH gene is located within an open reading frame (ORF) on human chromosome 16q22, spanning 1191 base pairs and encoding a 397‐amino acid protein [24]. Structurally, DHODH is characterized by two distinct domains connected by an extended loop: a smaller N‐terminal domain and a larger C‐terminal domain. The N‐terminal domain, comprising approximately 40 residues folded into two alpha‐helices (αA and αB) connected by a short loop, is associated with membrane binding [14]. In contrast, the cytosolic C‐terminal domain houses the redox‐active site, while the N‐terminal domain forms a membrane‐embedded tunnel that sequesters the flavin mononucleotide (FMN) binding site [25, 26]. Pyrimidine nucleotides are essential precursors for the biosynthesis of DNA, RNA, glycoproteins, and phospholipids, with cellular pools maintained through de novo and salvage pathways [25, 27]. DHODH serves as a rate‐limiting mitochondrial enzyme in de novo synthesis, catalyzing the oxidation of dihydroorotate to orotate in a redox reaction coupled with the reduction of CoQ to CoQH_2_ [12]. Subsequent pathway metabolites serve critical cellular functions: uridine diphosphate (UDP) acts as a donor for N‐acetylglucosamine (GlcNAc) in OGT‐mediated protein O‐GlcNAcylation, while deoxyuridine monophosphate (dUMP) is methylated to form deoxythymidine monophosphate (dTMP), a direct precursor for DNA synthesis [28, 29]. In addition to its metabolic role, DHODH is integral to the mitochondrial electron transport chain, where it is physically positioned between complexes II and III [30]. During the conversion of dihydroorotate to orotate, DHODH transfers electrons into the respiratory chain. Inhibition of DHODH therefore disrupts this electron supply, particularly under hypoxic or nutrient‐deprived conditions, leading to impaired function of complexes II and III and a consequent breakdown of oxidative phosphorylation [31, 32]. Through its catalytic activity, DHODH attenuates mitochondrial ROS generation, thereby suppressing ferroptosis and complementing the classical glutathione/GPX4 antioxidant pathway [20, 33]. In summary, DHODH is involved in a number of important physiological processes. Inhibitors targeting its enzyme activity sites can inhibit the proliferation, progression, or drug resistance of a variety of tumors [34, 35, 36].

The TRIM28 protein, also known as KRAB‐associated protein 1 (KAP1), is a 110 kDa multi‐domain member of the tripartite motif (TRIM) family, which comprises nearly 60 human proteins [37, 38]. The transcriptional repression mediated by TRIM28 follows a sequential mechanism: it is first recruited to DNA by KRAB‐ZNF proteins via its RBCC domain and undergoes auto‐SUMOylation [39, 40]. This SUMOylation enables the recruitment of SETDB1 and the NuRD complex to establish H3K9me3 marks and remove histone acetylation [41, 42]. The repressive state is then locked in place through HP1 binding, ensuring stable chromatin compaction and gene silencing [43]. In response to DNA double‐strand breaks (DSBs), TRIM28 plays a critical role in chromatin remodeling [44]. Upon DSB detection, the ATM kinase phosphorylates TRIM28 at Ser824, promoting chromatin decondensation to facilitate repair [45]. Loss of this phosphorylation impairs chromatin relaxation and increases cellular sensitivity to DSB‐inducing agents, while constitutive phosphorylation leads to persistent chromatin opening [46]. These findings establish TRIM28‐mediated chromatin relaxation as a central mechanism in the DNA damage response.

TRIM37, another key member of the human tripartite motif (TRIM) protein family, is encoded at the chromosomal locus 17q22‐q23 [47, 48]. This genomic region exhibits frequent amplification in multiple cancer types; notably, TRIM37 copy‐number gain is observed in approximately 50–60% of neuroblastomas and in over 10% of breast cancers [49, 50]. TRIM37 primarily functions as an E3 ubiquitin ligase, with its altered expression linked to tumorigenesis [47]. Studies have shown that TRIM37 knockdown suppresses tumor growth and Ras‐mediated Fas silencing, highlighting its role as a promoter of proliferation [51]. In breast cancer, elevated TRIM37 promotes histone H2 monoubiquitination, subsequently altering the expression of oncoproteins that drive progression [52]. Mechanistically, TRIM37 physically interacts with PRC2 subunits (EZH2/SUZ12), forming a complex that co‐occupies specific promoters to concurrently mediate H3K27 trimethylation and H2A monoubiquitination, thereby enforcing transcriptional repression [53, 54]. Beyond transcriptional regulation, TRIM37 rapidly translocates to the nucleus under genotoxic stress, where it interacts with TRAF6 to catalyze monoubiquitination of NEMO at K309, leading to NF‐κB activation and enhanced cell survival [55]. TRIM37 also facilitates epithelial‐mesenchymal transition in various cancer cell lines, suggesting a role in promoting metastasis [56, 57, 58].

VEGF is a critical downstream target of the HIF‐1 signaling pathway. The HIF‐1α/β heterodimer binds to hypoxia‐response elements (HREs), which contain the consensus sequence 5′‐ACGTG‐3′, in the regulatory regions of target genes [59, 60]. This binding drives the transcription and translation of pro‐angiogenic factors such as VEGF and PDGF, thereby promoting tumor angiogenesis [10, 61]. Antiangiogenic agents, including sunitinib and sorafenib, were developed to target this pathway and have been shown to markedly reduce tumor vascularization and suppress tumor growth [11]. This study focused on the classic VEGFA pathway and uncovered a novel mechanism through which DHODH drives sunitinib resistance in renal cell carcinoma via a non‐enzymatic function. While activation of the VEGF pathway is known to be a key contributor to sunitinib resistance, our findings further demonstrate that, in resistant models, DHODH directly interacts with the transcription factor TRIM28 and competitively inhibits its ubiquitination at lysine 366 by the E3 ligase TRIM37, thereby stabilizing the TRIM28 protein. The stabilized TRIM28 subsequently transcriptionally activates VEGFA, reactivating the VEGF signaling pathway and ultimately leading to sunitinib treatment failure. Notably, this resistance mechanism is independent of DHODH's enzymatic activity, suggesting that conventional inhibitors targeting its catalytic site may be insufficient to reverse resistance. Based on this insight, we screened for small‐molecule compounds that specifically disrupt the DHODH–TRIM28 interaction and identified Lisaftoclax as a promising candidate. Lisaftoclax is a B‐cell lymphoma 2 (BCL‐2) inhibitor developed by Ascentage Pharma for the treatment of haematological malignancies [62]. It selectively binds BCL‐2 (Ki < 0.1 nmol/L), disrupts BCL‐2:BIM complexes, and compromises mitochondrial outer membrane potential, culminating in BAX/BAK‐dependent, caspase‐mediated apoptosis [63]. Experimental validation showed that Lisaftoclax effectively blocks the DHODH–TRIM28 binding, reduces TRIM28 and VEGFA levels, and restores tumor sensitivity to sunitinib in vitro, in patient‐derived organoids, and in vivo models.

While this study provides compelling evidence for a novel mechanism of DHODH‐mediated sunitinib resistance and identifies a promising therapeutic strategy, several limitations should be acknowledged. First, the mechanistic insights were primarily derived from in vitro models and patient‐derived xenografts. Although these systems are highly relevant, they cannot fully recapitulate the complexity of the human tumor microenvironment, immune interactions, and systemic drug metabolism. Future studies should validate these findings in additional in vivo models and, ultimately, in clinical samples from patients who developed resistance during sunitinib treatment. Second, the therapeutic efficacy of Lisaftoclax was demonstrated in preclinical models. Its pharmacokinetics, safety profile, and optimal dosing schedule in humans remain to be determined. Furthermore, the potential off‐target effects and long‐term consequences of disrupting the DHODH‐TRIM28 interaction require thorough investigation.

This work opens a new avenue for overcoming sunitinib resistance in RCC. Future research should focus on translating these preclinical findings into clinical trials, comprehensively evaluating the safety and efficacy of Lisaftoclax in combination with sunitinib, and further elucidating the broader biological network governed by the non‐enzymatic functions of DHODH.

## 5 Conclusion

In conclusion, DHODH drives sunitinib resistance in RCC through a non‐enzymatic mechanism by competing with TRIM37, stabilizing TRIM28, and activating VEGFA transcription. Targeting this interaction with lisaftoclax restores sunitinib sensitivity, offering a promising therapeutic strategy.

## Author Contributions

S.Q. carried out the investigation, curated the data, performed the visualization, contributed to conceptualization, and drafted the original manuscript. S.P. curated and validated the data, and reviewed and edited the manuscript. Z.X. contributed resources and curated the data. Y.D. curated the data. L.J. performed visualization. B.L. curated the data. Q.L. contributed to methodology. Z.W. supervised the study and acquired funding. J.X. contributed to methodology and supervised the study. A.X. supervised the study, administered the project, and acquired funding. All authors read and approved the final manuscript.

## Funding

This work was supported by Jiangsu Provincial Medical Key Discipline (Laboratory) (ZDXK202219), Young Scholars Fostering Fund of the First Affiliated Hospital of Nanjing Medical University (PY2023040), National Natural Science Foundation of China (82403422), Natural Science Foundation of Jiangsu Province (BK20241127), and the China Postdoctoral Science Foundation (2024M761480).

## Ethics Statement

All clinical renal cell carcinoma tissue specimens were obtained from patients at First Affiliated Hospital of Nanjing Medical University. Informed consent was obtained from each participant prior to sample collection and all patients provided informed consent for participating in this study. The collection and analysis of tumor and clinical data were approved by the Ethics Committee of Nanjing Medical University (approval No. 2021‐SR‐430), and the research adhered to principles outlined in the Declaration of Helsinki. Clinical trial number: not applicable. Animal studies were conducted following approval from the Institutional Animal Care and Use Committee (IACUC) of Nanjing Medical University (approval No. 2406068). The construction of the xenotransplantation model complied with institutional guidelines and received approval from the IACUC of Nanjing Medical University.

## Consent

All the authors consent for this manuscript publication.

## Conflicts of Interest

The authors declare no conflicts of interest.

## Supporting information




**Supporting File**: advs76266‐sup‐0001‐SuppMat.docx.

## Data Availability

The data that support the findings of this study are available from the corresponding author upon reasonable request.
