# Supplementary material for: DHODH Drives Sunitinib Resistance Via a Non‐Enzymatic Mechanism by Inhibiting TRIM28 Ubiquitination and Consequent VEGFA Activation in RCC
Source: Adv Sci (Weinh). 2026 Jun 23;13(52):e76266. doi: 10.1002/advs.76266 (PMC13337078; doi:10.1002/advs.76266)
Supplement: Supplementary file 1 [file ADVS-13-e76266-s001.docx]

**Supplement Table S1.** shRNA sequences in this study

| Name | Sequence (5’-3’) |
| --- | --- |
| ShDHODH-1 | TCCGGGATTTATCAACTCAAA |
| ShDHODH-2 | GTGAGAGTTCTGGGCCATAAA |
| ShTRIM28-1 | GAGAATTATTTCATGCGTGAT |
| ShTRIM28-2 | GAGGACTACAACCTTATTGTT |
| ShSUMO2-1 | GACTGAGAACAACAATCATAT |
| ShSUMO2-2 | CCTATTGTGAACGACAGGGAT |
| ShTRIM37-1 | GCTGAAGAATAAGCTTATA |
| ShTRIM37-2 | GCTACGAGAACTAGTAAAT |

**Supplement Table S2.** Sequence(5'to3') of the primers for qRT-PCR.

| Gene | Forward | Reverse |
| --- | --- | --- |
| DHODH | CCACGGGAGATGAGCGTTTC | CAGGGAGGTGAAGCGAACA |
| TRIM28 | TTTCATGCGTGATAGTGGCAG | GCCTCTACACAGGTCTCACAC |
| VEGFA | AGGGCAGAATCATCACGAAGT | AGGGTCTCGATTGGATGGCA |
| VEGFA-CUT&RUN-1 | CCCTTTCCAAAGCCCATTC | ATTCACCCAGCTTCCCTGTG |
| VEGFA-CUT&RUN-2 | CTGGTAGCGGGGAGGATC | GCCACGACCTCCGAGCTA |
| VEGFA-CUT&RUN-3 | GGAAGCATCCCTGGACACTT | CCTGCAGACATCAAAGTGAGC |
| GAPDH | GGAGCGAGATCCCTCCAAAAT | GGCTGTTGTCATACTTCTCATGG |

**Supplement Table S3.** Antibodies used in this study.

| Anitbody | Source | Cat.No. |
| --- | --- | --- |
| Rabbit DHODH | Proteintech | Cat#14877-1-AP |
| Rabbit TRIM28 | Abclonal | Cat#A19568 |
| Mouse TRIM28 | Proteintech | Cat#66630-1-Ig |
| Rabbit VEGFA | Proteintech | Cat#81323-2-RR |
| Rabbit TRIM37 | Proteintech | Cat#13037-1-AP |
| Rabbit TRIM21 | Abclonal | Cat#A22724 |
| Rabbit Caspase | Proteintech | Cat#19677-1-AP |
| Rabbit Cleaved Caspase-3 | CST | Cat#9661 |
| Rabbit Ubiquitin | CST | Cat#20326 |
| Rabbit SUMO2 | Abclonal | Cat#A22734 |
| Rabbit Flag | CST | Cat#14793 |
| Rabbit His | CST | Cat#12698 |
| Rabbit GFP | CST | Cat#2555 |
| Mouse Alpha Tubulin | Proteintech | Cat#11224-1-AP |
| HRP-conjugated Goat Anti-Rabbit IgG | Proteintech | Cat#SA00001-2 |
| HRP-conjugated Goat Anti-Mouse IgG | Proteintech | Cat#SA00001-1 |
| Mouse+Rabbit Universal AntiBody Heavy Chain + Light Chain Secondary Antibody | Abmart | Cat#M21009 |
| Goat Anti-Mouse IgG H&L (Alexa Fluor® 594) | Abcam | Cat#ab150116 |
| Goat Anti-Rabbit IgG H&L (Alexa Fluor® 488) | Abcam | Cat#ab150077 |
